# Context-Specific Reweighting of Auditory Spatial Cues following Altered Experience during Development

**DOI:** 10.1016/j.cub.2013.05.045

**Published:** 2013-07-22

**Authors:** Peter Keating, Johannes C. Dahmen, Andrew J. King

**Affiliations:** 1 Department of Physiology, Anatomy and Genetics, University of Oxford, Parks Road, Oxford OX1 3PT, UK

## Abstract

**Background:**

Neural systems must weight and integrate different sensory cues in order to make decisions. However, environmental conditions often change over time, altering the reliability of different cues and therefore the optimal way for combining them. To explore how cue integration develops in dynamic environments, we examined the effects on auditory spatial processing of rearing ferrets with localization cues that were modified via a unilateral earplug, interspersed with brief periods of normal hearing.

**Results:**

In contrast with control animals, which rely primarily on timing and intensity differences between their two ears to localize sound sources, the juvenile-plugged ferrets developed the ability to localize sounds accurately by relying more on the unchanged spectral localization cues provided by the single normal ear. This adaptive process was paralleled by changes in neuronal responses in the primary auditory cortex, which became relatively more sensitive to these monaural spatial cues. Our behavioral and physiological data demonstrated, however, that the reweighting of different spatial cues disappeared as soon as normal hearing was experienced, showing for the first time that this type of plasticity can be context specific.

**Conclusions:**

These results show that developmental changes can be selectively expressed in response to specific acoustic conditions. In this way, the auditory system can develop and simultaneously maintain two distinct models of auditory space and switch between these models depending on the prevailing sensory context. This ability is likely to be critical for maintaining accurate perception in dynamic environments and may point toward novel therapeutic strategies for individuals who experience sensory deficits during development.

## Highlights

• Ferrets reared with a unilateral hearing loss are able to localize sounds accurately
• Adaptation relies on cue reweighting that reverses when normal hearing is available
• Auditory cortical neurons show corresponding context-specific plasticity
• Contextual cue reweighting maintains perceptual stability in dynamic environments

## Graphical Abstract

**Figure:**
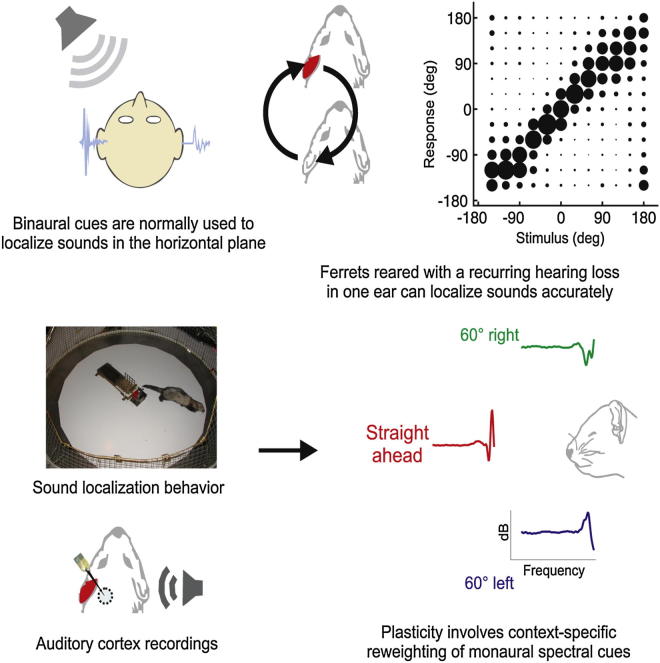

## Introduction

Although experience is critical for shaping the way sensory systems develop, it is unclear how they respond to environmental conditions that change over time. In the case of spatial hearing, developmental plasticity has been studied primarily by inducing a stable hearing loss in one ear, a manipulation that alters the binaural cues used for sound localization [1–8]. This work shows that abnormal acoustical experience during early life can produce profound changes in neuronal sensitivity to those cues, which typically persist even when normal hearing is provided at a later stage. Thus, although potentially helping to localize sounds accurately in the presence of a unilateral hearing loss [1–3], this plasticity can become maladaptive, resulting in impaired spatial processing, once normal inputs are available. In mammals, this disruption of normal spatial hearing is thought to reflect “amblyaudia,” a condition in which there is a weakening of the input provided by the developmentally deprived ear [6].

It is unclear, however, what happens when the developing auditory system experiences an intermittent hearing loss in one ear. This is particularly relevant to the development of spatial hearing, since recurring periods of hearing loss are extremely common in infancy [9, 10]. From a clinical perspective, it is important to know whether amblyaudia can be reversed by brief periods of normal hearing, or whether spatial hearing is instead degraded further by acoustical inputs that change repeatedly over time. A more fundamental issue, however, concerns how sensory systems adapt to dynamic environments that switch between distinct states but are locally stable in time. In this respect, a recurring hearing loss provides an excellent experimental model for studying a much wider class of problem faced by the brain.

One solution to this problem would be to rely more on cues that remain unchanged over time. Under normal hearing conditions, the location of a sound in the horizontal plane is determined primarily by interaural level differences (ILDs) and interaural time differences (ITDs) [11, 12], both of which are disrupted by hearing loss in one ear. In addition to these binaural spatial cues, however, mammals possess an external ear that filters high-frequency sounds in a direction-dependent manner, generating monaural spectral cues for sound location [12–14]. Since a unilateral hearing loss has no effect on the spectral cues provided by the intact ear, the auditory system could adapt during development by relying more on these monaural spatial cues. Such cue reweighting would presumably have a profound impact on how sounds are localized, even when normal hearing is made available. Alternatively, the auditory system could acquire and maintain different models of the external world and switch between these models depending on the prevailing sensory context.

To address this issue, we reared ferrets with a recurring hearing loss in one ear and obtained behavioral and neurophysiological measures of spatial processing from the same animals. Our results demonstrate that the auditory system can indeed switch between different strategies for localizing sound depending on the sensory context.

## Results

### Behavioral Adaptation to an Earplug

**Figure 1:**
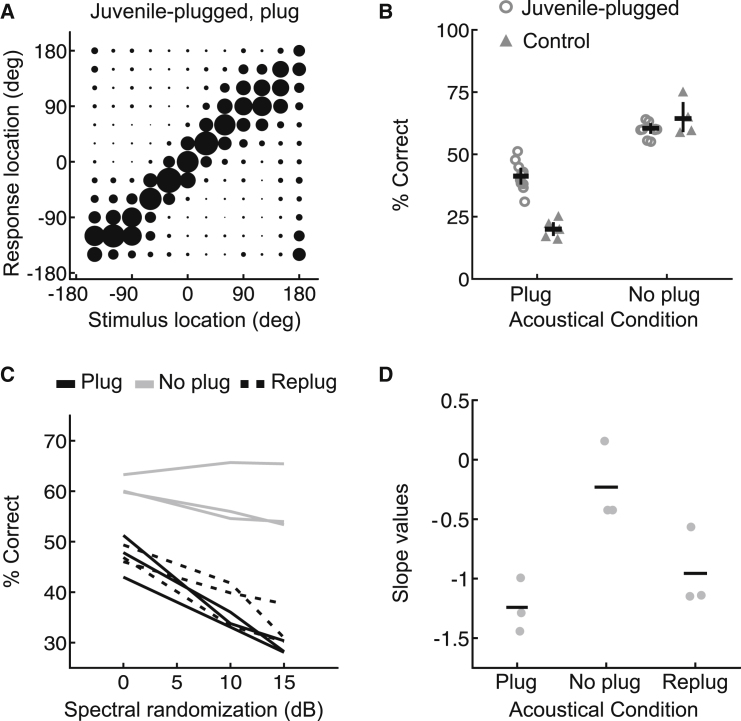
Adaptive Changes in Sound Localization following Developmental Hearing Loss in One Ear (A) Average joint distributions of stimulus and response location for juvenile-plugged ferrets wearing an earplug; the size of the circles represents the proportion of trials for each stimulus-response combination. (B) Percentage correct scores for these groups, with individual animals denoted by symbols. Horizontal lines indicate mean values, with error bars showing bootstrapped 95% confidence intervals. (C) Effect of degree of spectral randomization on localization by juvenile-plugged animals with the ear plugged (solid black lines), unplugged (solid gray lines), or replugged (dashed black lines). (D) Slope values derived from (C), with mean slope values shown by horizontal lines. See also Figure S1.

We reared ten animals from the age of hearing onset with an earplug in the left ear but allowed them relatively brief, intermittent periods of normal hearing. Earplugs delay and attenuate sounds in a frequency-dependent manner [15–17], altering the spatial cue values corresponding to each direction in space while preserving the monaural spatial cues available to the nonoccluded ear. Whereas acutely plugging one ear in normally reared adult ferrets severely degraded sound localization accuracy (Figure 1B; see also Figures S1A and S1B available online), juvenile-plugged ferrets performed the same task significantly more accurately while wearing an earplug (p < 0.001; bootstrap test; Figures 1A and 1B), indicating that some form of adaptation had taken place. Following earplug removal, we observed no difference between juvenile-plugged and control animals (p = 0.56; bootstrap test), with each group of animals performing better than when one ear was occluded (p < 0.001, bootstrap test; Figures 1B, S1C, and S1D). Thus, although juvenile-plugged ferrets largely adapt to the presence of the earplug, this does not interfere with their ability to localize sounds when they are provided with normal hearing. Response times in juvenile-plugged animals wearing an earplug were additionally very similar to those obtained from controls under normal hearing conditions (p > 0.05; bootstrap test), suggesting that the adaptive mechanism does not require any additional processing time.

We also measured localization performance in three juvenile-plugged animals using broadband stimuli in which spectral cues were disrupted by introducing varying amounts of spectral randomization, a manipulation that has a negligible effect on the accuracy of azimuth judgments by human listeners with normal binaural hearing but that should make monaural localization much more difficult [16, 18]. In the presence of an earplug, these animals performed progressively less well as the amount of spectral uncertainty was increased (significant difference in % correct between 0 and 15 dB randomization conditions; p < 0.001; mixed-effects logistic regression, post hoc test; Figures 1C and S1E). This can be seen in the negative slope of the relationship between task performance and degree of spectral randomization (Figure 1D) and indicates that sound localization by animals that had adapted to a unilateral hearing loss was dependent on spectral cues.

If juvenile-plugged animals rely solely on spectral cues to localize sound, then their susceptibility to spectral randomization should remain when the earplug is removed. Alternatively, if these animals can switch between different cues, then randomization should have little effect once balanced binaural inputs are provided. Consistent with this latter hypothesis, we found that the effects of spectral randomization were much less pronounced without an earplug (significant interaction between degree of randomization and presence/absence of an earplug; p < 0.001; mixed-effects logistic regression; Figures 1D and S1F), and were not significantly different from zero (no difference in performance across different randomization conditions; p > 0.05; post hoc tests). Strong effects of spectral randomization returned, however, when an earplug was reintroduced (significant difference in % correct between 0 and 15 dB randomization conditions; p < 0.001; mixed-effects logistic regression, post hoc test; Figures 1C and 1D). Thus, the cue reweighting we observe not only is dependent on the animals’ early experience but also is specific to situations in which a unilateral hearing loss is present.

### Recovering Spectral Cues Using Reverse Correlation

In principle, adaptation could be achieved by the auditory system becoming more dependent on the preserved spectral cues available to the unaffected ear and less on the spectral cues provided to the occluded ear. To test this, we exploited the fact that monaural listeners find it extremely difficult to dissociate the spectral properties of the source stimulus from those imparted by the filtering effects of the head and ears. Indeed, when localizing sounds in the vertical plane, individuals may experience illusory spatial percepts that result in predictable errors if the source stimulus contains features that mimic the spectral cues associated with a particular location [19–21].

**Figure 2:**
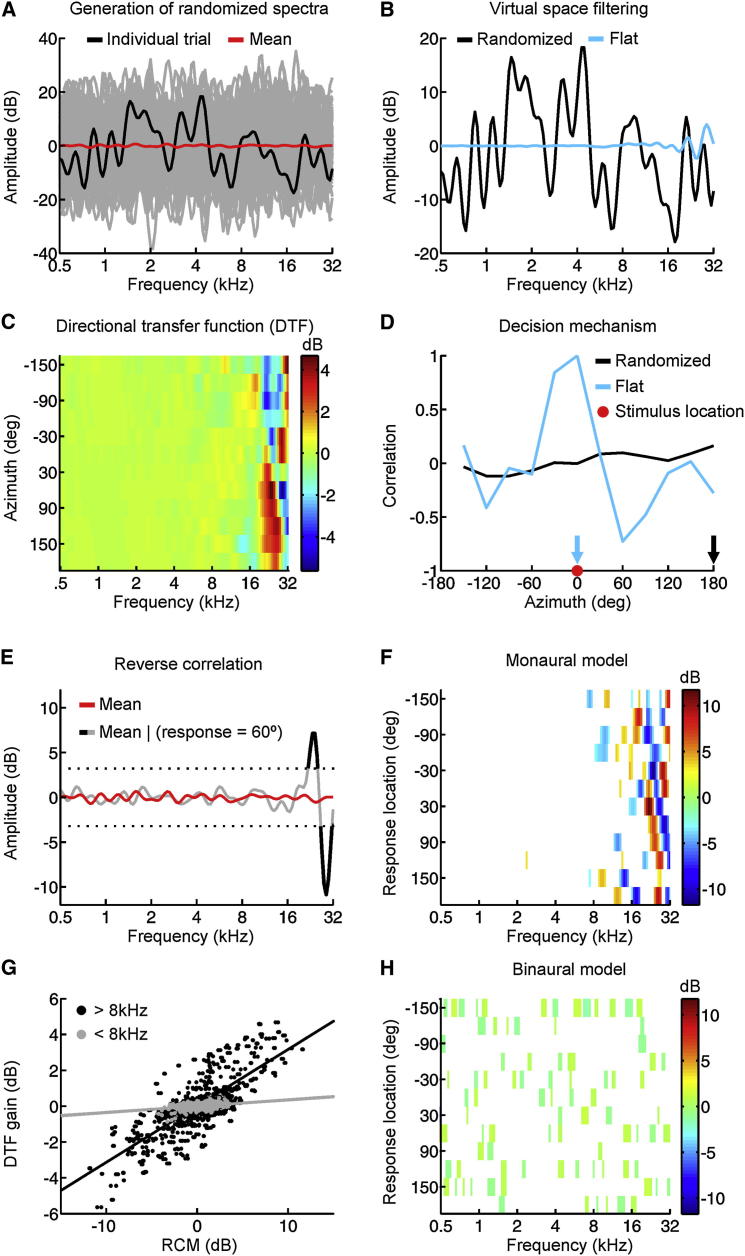
Characterizing Dependence on Spectral Cues in a Model of Sound Localization (A) Stimuli with randomized spectra (gray). Amplitude varied considerably across frequency on a single trial (black), but the mean spectrum across trials was relatively flat (red). (B) On each trial, a stimulus was presented at one of 12 virtual locations, which was achieved by convolving the source stimulus with the external ear’s transfer function appropriate for that location. Examples of flat (blue) and randomized-spectrum (black) stimuli convolved with the ear’s transfer function (DTF) for 0° azimuth. (C) Directional transfer function (DTF) used by the monaural model to infer sound location, derived from acoustical measurements from a single ferret. This illustrates the spectral localization cues generated in the horizontal plane by the immobile right external ear of this individual. Warmer colors indicate higher gains. (D) In order to determine its response, the model computed the correlation between the spectrum of the spatially filtered stimulus and the ear’s transfer function associated with each of the 12 possible locations (C), identified the transfer function that was maximally correlated with the stimulus, and selected the corresponding location as its response (denoted by arrows). The correlation between the amplitude spectra of stimuli corresponding to a virtual azimuth of 0° and the transfer functions associated with different locations is shown for stimuli with flat (blue) and randomized (black) source spectra. (E) The mean spectrum associated with each response location (gray/black line) typically differed from the overall mean across all locations (red). Data are shown for trials on which the model responded to a stimulus location of 60°. (F) Reverse correlation map (RCM) constructed by thresholding (±1.5 SD) the mean spectra shown in (E). Colors show differences between the overall mean and the mean stimulus associated with each response location. (G) Degree of similarity between the RCM in (F) and the gain of the DTF shown in (C), as determined by applying linear regression to the point-wise comparison between the two measures. Each dot shows a particular combination of frequency and location. Data corresponding to high (>8 kHz; black) and low (<8 kHz; gray) frequencies are plotted separately. (H) RCM obtained using a binaural model.

Based on reverse correlation, an approach often used to reveal the basis for spatial sensitivity in auditory cortical neurons [22, 23], we developed a method to determine whether specific features of the source spectrum consistently elicit behavioral responses to a particular location. To ensure that this approach could be successfully applied to sound localization behavior, we first constructed a simple model of monaural sound localization and simulated the task described in Figure 1 (Figures 2A–2D). Although this model accurately localized stimuli with flat spectra, randomization of the source spectrum typically produced errors in sound localization, with the nature of those errors determined by the spectral properties of the source stimulus.

Using the simulated data obtained from 5,000 trials, we extracted the set of trials that the model attributed to a particular location and identified the mean stimulus presented on those trials (Figure 2E). This process was repeated for each response location in order to construct a reverse correlation map (RCM), to which a threshold (±1.5 SD) was applied (tRCM), thereby reducing random variability in the data (see Supplemental Experimental Procedures). The resulting tRCM revealed clear high-frequency features that varied as a function of response location (Figure 2F). Importantly, the features present in the RCM were positively correlated with the gain of the external ear’s directional transfer function (DTF) (Figure 2C) used to infer source location by the underlying model (Figure 2G).

For comparison purposes, we also constructed a binaural version of the model that inferred sound location on the basis of frequency-dependent ILDs. In contrast to the monaural model, the tRCM produced by the binaural model contained no discernible features that varied with response location (Figure 2H), and the features that did emerge were much smaller in amplitude, reflecting the fact that spatial information provided by ILD cues is independent of the source spectrum. This method therefore provides an additional way of distinguishing between reliance on monaural and binaural cues.

### Behavioral Reweighting of Spectral Cues

**Figure 3:**
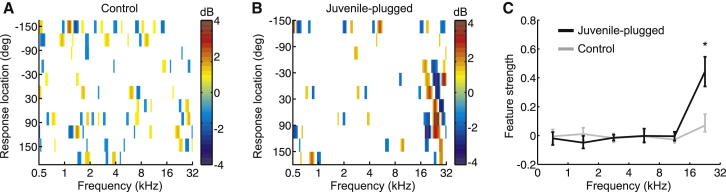
Characterizing the Dependence of Sound Localization Behavior on Spectral Cues at Different Sound Frequencies (A) RCM for a single control animal. Color shows differences between the overall stimulus mean and the mean stimulus at each response location. (B) RCM for a single juvenile-plugged animal. (C) Strength of correlation between stimulus spectrum and response, as quantified by the “feature strength,” which measures the mean amplitude of features that emerge in the RCM as a function of frequency (see Supplemental Experimental Procedures). Lines show mean feature strength for juvenile-plugged (black) and control (gray) animals, with error bars denoting bootstrapped 95% confidence intervals.

The modeling described in Figure 2 shows that reverse correlation can, in principle, be used to measure sensitivity to monaural spectral cues within the context of a localization task. We therefore employed this method to assess the importance of these cues for sound localization by ferrets. To obtain a baseline measure of sensitivity, we first computed a separate RCM for each of the normally reared controls under conditions of normal hearing. As was the case for the binaural model described above (Figure 2H), the RCMs obtained from controls failed to show any obvious spectral features that varied with location (Figure 3A), suggesting that their localization responses are largely independent of the spectral content of the stimuli. In keeping with our monaural model of sound localization (Figure 2F), however, the RCMs obtained from juvenile-plugged ferrets wearing an earplug contained distinctive high-frequency features that changed gradually as a function of response location (Figure 3B).

To quantify the frequency dependence of these spectral features, we estimated the “feature strength” for each animal, a measure that produced positive values for frequency bands in which spectral features emerged (see Supplemental Experimental Procedures). In control animals, the feature strength values were small and relatively constant across frequency (Figure 3C), only achieving values that were significantly >0 for the highest-frequency band tested (p < 0.001; bootstrap test). The feature strength values in juvenile-plugged ferrets showed a similar overall pattern, but were significantly greater than in controls for the high frequencies from 16 to 32 kHz (p < 0.001; bootstrap test; Figure 3C) that contain most of the spectral features that aid monaural localization. Thus, while the localization responses of both groups showed some sensitivity to high-frequency features of the source stimulus, this effect was much greater in ferrets that had adapted to an earplug.

**Figure 4:**
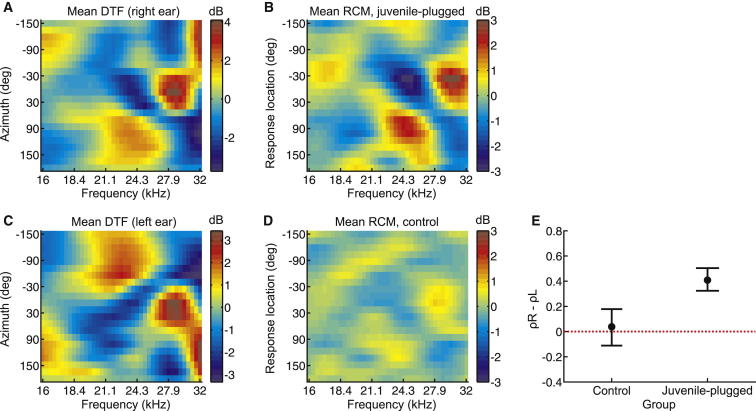
Dependence of Sound Localization Behavior on the Spectral Cues Provided by Each Ear (A) Right-ear DTFs averaged across animals. Data have been interpolated for visualization purposes. (B) Interpolated RCM averaged across juvenile-plugged animals. (C) Interpolated left-ear DTFs averaged across animals. (D) Interpolated RCM averaged across control animals. (E) Difference in correlation between the RCM and the mean right ear DTF (ρR) and between the RCM and the mean left ear DTF (ρL). Positive values indicate that ρR is greater than ρL. Error bars show bootstrapped 95% confidence intervals. A much higher correlation was found for the right, nonoccluded ear in the ferrets that were reared with the left ear plugged, indicating greater dependence of localization responses on those spectral cues, whereas no differences between the ears were observed in the control animals.

In our model of monaural localization (Figure 2), the features revealed by reverse correlation reflected the cues used by the underlying model to infer sound source location. Therefore, if our juvenile-plugged ferrets were relying more on the preserved spectral cues available to their open ear, we might expect the features that emerge in the RCM to correlate with these cues. We restricted this analysis to the 16–32 kHz range where the feature strength values were significantly higher than in controls. This also matches the frequency range over which the directional filtering effects of the ferret head and ears produce spectral features that shift linearly in center frequency with azimuth, as illustrated by the DTFs measured both for individual animals (dark blue and orange regions in Figure 2C) and averaged across a database of comparable animals (Figure 4A) [24].

We therefore averaged the RCM data across juvenile-plugged animals (Figure 4B) and computed the partial correlation between the resulting mean RCM and the mean DTF measured for the right (ρR; Figure 4A) and left ears (ρL; Figure 4C), controlling in each case for the effect of the other ear. Because we were interested in the *relative* importance of the two ears, we then calculated the difference between ρR and ρL, where positive values indicated a dominance of right-ear spectral cues. For comparison purposes, we also averaged the RCM data across control animals (Figure 4D) and repeated the same analyses.

Whereas the mean RCM obtained from controls was equally correlated with the DTFs provided by each ear (i.e., the difference between ρR and ρL was close to zero; Figure 4E), the mean RCM obtained from juvenile-plugged animals showed a significantly higher correlation with the right-ear DTFs (subtracting ρL from ρR produced positive values; p < 0.001; bootstrap test; Figure 4E). Thus, the spectral features that influence the behavioral responses of juvenile-plugged animals primarily reflect the preserved monaural spectral cues available at the nonoccluded ear, suggesting that these animals have become more dependent on these cues for localizing sounds and less on the cues normally available to the plugged ear. This does not mean, however, that controls ignore spectral features altogether when localizing sounds in azimuth, as we found a significant correlation between the mean RCM and the mean DTF for both the left (r = 0.35; p < 0.001) and right (r = 0.42; p < 0.001) ears. Adaptation to a unilateral hearing loss during development therefore entails changing the relative weighting of those cues in opposite directions for each ear.

### Cortical Reweighting of Spectral Cues

**Figure 5:**
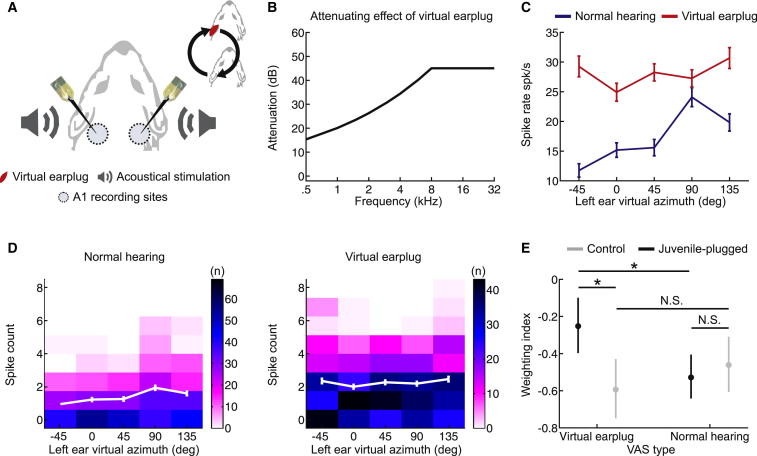
Cortical Reweighting of Auditory Spatial Cues (A) Setup used for bilateral A1 recordings, with recording sites illustrated as light blue circles. Virtual acoustic space stimuli were presented to both ears and alternated between conditions that recreated normal spatial cues or a virtual earplug in the left ear (red). (B) Frequency-dependent attenuation by the virtual earplug. (C) Azimuth response profile (mean rate ± SEM) for an A1 unit that was sensitive to the virtual earplug is plotted for both normal hearing (blue) and left virtual earplug (red) conditions. The virtual azimuth angles correspond to the left-ear spectral cue values associated with each location, with the data collapsed across the spatial cues values provided by the other ear. (D) Joint distributions of spike count and left-ear virtual azimuth for the unit shown in (C), with colors indicating the number of trials for each virtual-azimuth-spike-count combination. Azimuth response profiles (mean count ± SEM) are plotted in white. (E) Weighting index values (mean ± 95% confidence intervals) for all driven units in juvenile-plugged (black) and control (gray) animals. Higher values indicate that more weight was given to the spatial cues provided to the intact ear.

In order to understand the neural mechanisms underlying this form of experience-dependent plasticity, we performed bilateral extracellular recordings from a mixture of single units and small multiunit clusters in the primary auditory cortex (A1) of both juvenile-plugged ferrets and controls (Figure 5A). Because the behavioral plasticity produced by monaural deprivation was observed only at the highest frequencies tested (Figure 3C) and because spectral cues in this species are largely uninformative with respect to spatial location below 8 kHz (Figures 2C, 2F, and 2G), we focused on neurons with a characteristic frequency (CF) > 8 kHz. To determine whether high-frequency A1 neurons in juvenile-plugged animals become more sensitive to the monaural spatial cues provided to the intact ear and less sensitive to the other available cues, specifically ILDs and the monaural spatial cues available at the other ear, we used virtual acoustic space (VAS) techniques to simulate over headphones either normal free-field listening conditions or an earplug in the left ear (Figure 5B). This was done in an attempt to reproduce the acoustical inputs experienced by the juvenile-plugged ferrets. Using VAS stimuli enabled us to manipulate auditory spatial cues independently of one another and therefore to assess neuronal sensitivity to each cue separately.

We constructed tuning curves by measuring the effect of changing the value of individual cues on the mean firing rate of the neurons. Introducing a virtual earplug dramatically altered the tuning curves for some of these units (Figure 5C) but had very little impact on others. To quantify the sensitivity of A1 neurons to different cues, we counted the number of spikes that occurred on each trial within a time window that spanned the neural response and used these data to produce joint distributions of spike count and cue value (Figure 5D). The observed spike count distributions were then used to estimate the mutual information (MI) between spike count and a particular cue [25].

We next constructed a weighting index that compared the MI values obtained using the monaural spatial cues provided to the nonoccluded right ear with the sum of the MI values obtained using the other available spatial cues. The index was designed so that its value would increase if a neuron were to become relatively more sensitive to the right-ear monaural spatial cues. Consequently, our behavioral data predict that this neural weighting index should be higher in juvenile-plugged animals than in controls, but only when a virtual earplug is experienced.

Our A1 data conformed to precisely this pattern (Figure 5E). Thus, prior experience of an earplug determined how the weighting index changed when a virtual earplug was introduced (interaction between group and presence/absence of the virtual earplug; p = 0.0014; permutation test). This is because A1 neurons in juvenile-plugged animals had higher weighting index values than those in controls whenever a virtual earplug was present (p < 0.05; post hoc test), but this difference disappeared under conditions of normal hearing. This shows that cortical neurons recorded from juvenile-plugged animals are relatively more sensitive to the intact monaural spatial cues provided by the nonoccluded ear and less sensitive to the other available spatial cues. Although the weighting index values indicate that cortical neurons in these animals still carry more information about the other available cues than about the monaural spectral cues provided by the nonoccluded ear, the increased reliance on the spectral cues appears to be critical for their ability to localize sounds while they are wearing an earplug. These neurophysiological changes are specific, however, to a particular sensory context. Thus, when normal hearing is available, we found no difference in cue sensitivity between groups, whereas the juvenile-plugged animals rapidly switch to relying relatively more on the intact set of monaural spatial cues whenever a virtual earplug is experienced.

As predicted by our behavioral (Figure 3) and modeling (Figures 2F and 2G) data, this neurophysiological reweighting was specific to neurons tuned to high frequencies, since we found no evidence for experience-dependent reweighting in units with CFs < 8 kHz (interaction between group and presence/absence of the virtual earplug; p = 0.21; permutation test). Indeed, experience-dependent plasticity was significantly greater in units with CFs > 8 kHz than in those with CFs < 8 kHz (interaction between high/low CF, group, and presence/absence of virtual earplug; p = 0.0004; permutation test). Moreover, by analyzing the data obtained from high-frequency units separately for each hemisphere, we found that prior experience of an earplug primarily affects the cortex ipsilateral to the developmentally plugged left ear, producing context-dependent changes in the processing of monaural spatial cues whenever a virtual earplug was introduced in that ear (interaction between group and presence/absence of the virtual earplug: left-ear spatial cues, p = 0.021; right-ear spatial cues, p < 0.001; permutation test). Relative to controls, neurons in this hemisphere of juvenile-plugged ferrets showed less sensitivity to the monaural spatial cues provided by the developmentally occluded ear (p < 0.05; post hoc test) and greater sensitivity to the monaural cues provided by the contralateral, nonoccluded ear (p < 0.05; post hoc test). Again, these changes were limited to situations in which a virtual earplug was experienced. In contrast, no effects of prior experience were observed in the right hemisphere, contralateral to the developmentally plugged ear, with juvenile-plugged animals and controls showing very similar MI values across all conditions.

## Discussion

Our goal in this study was to understand how spatial hearing develops when ferrets experience acoustical inputs that change over time, focusing in particular on the effects of an intermittent hearing loss in one ear. In contrast to the poor performance of acutely plugged controls, ferrets reared with one ear occluded acquired the ability to localize sounds accurately while wearing an earplug, and did so by becoming more dependent on the unchanged spectral cues provided by the intact ear. Surprisingly, however, when these animals were subsequently provided with normal inputs to each ear, they became indistinguishable from controls. The mechanism of experience-dependent adaptation therefore appears to involve a context-specific reweighting of different cues, which allows accurate sound localization to be maintained under both normal and abnormal hearing conditions.

These findings contrast with the results of previous developmental studies of unilateral hearing loss, which show pronounced changes in spatial processing following the restoration of normal hearing [1–8]. One important difference between earlier work and the experiments reported here, however, concerns the method of inducing hearing loss. Whereas previous studies induced a stable hearing loss in one ear, we used earplugs that were periodically replaced over time, thereby providing our animals with limited exposure to normal acoustical input. Since persistent changes in the processing of spatial cues have been observed following stable monaural deprivation during infancy, even in cases where normal inputs were available for some time prior to the onset of hearing loss [6, 26], our results suggest that intermittent experience of normal hearing may be important for preserving sensitivity to those cues. This is consistent with studies of monocular deprivation, which show that relatively short periods of normal experience may be enough to preserve the input provided by the deprived eye [27]. The present results therefore suggest that similar principles may apply to amblyaudia, which has important implications for auditory processing deficits arising from hearing loss during development [9, 10, 28].

If the shape of their external ears is modified by wearing molds, adult humans can learn to unambiguously map auditory space onto two different sets of spectral cues [14]. Our results complement this finding by showing that the developing auditory system can acquire the ability to weight the *same* cues in different ways depending on the acoustical context and provide the first evidence linking this type of behavioral plasticity with changes in neuronal responses, in particular those of cortical neurons located in the hemisphere ipsilateral to the affected ear. Although it is conceivable that alternative weighting strategies might be implemented by different sets of neurons, recent work suggests that multiple sensory cues can be multiplexed onto the same sets of neurons [29], suggesting that separate neuronal populations may not be required to implement different weighting strategies.

Previous studies have shown that when perceptual judgments are based on cues from different sensory modalities, the greatest weight is given to those cues that provide the most reliable information [30, 31]. To the extent that auditory spatial cue reliability is affected by the presence or absence of a unilateral earplug, our results are consistent with theories of optimal cue integration. Importantly, however, the reweighting we observed is not fully explained by the acoustical context itself but also depends on prior experience of that context. Thus, while some reweighting may take place as soon as the cues are altered by occluding one ear [32], our data show that longer-term experience of the altered cues results in greater dependence on the intact spectral cues without compromising the set of weights that are deployed when balanced binaural hearing is possible. The auditory system can therefore acquire the ability to deploy different weighting strategies according to the sensory conditions experienced, which helps to maintain accurate sound localization in spite of pronounced variations in inputs.

Although reweighting of spectral cues represents a useful strategy for adapting to changes in auditory inputs, adaptation can also be achieved by remapping altered binaural cues onto appropriate spatial locations. Indeed, this is the way developing barn owls compensate for the effects of monaural deprivation [2, 3, 33]. In contrast, recording studies in mammals raised with a stable unilateral hearing loss have found no evidence for adaptive shifts in binaural sensitivity and have even observed shifts that are maladaptive [4–8]. However, while the external ear structures of the barn owl are directionally sensitive [34], these animals do not have access to the high-frequency spectral cues that are available to mammals. By demonstrating the importance of these high-frequency spectral cues for adaptation in ferrets, our results help to reconcile the barn owl data with those obtained in mammals. Thus, whereas mammals can use high-frequency intact monaural spectral cues, barn owls may be forced to reinterpret abnormal binaural cues.

Although previous work has emphasized the importance of spectral cues for vertical localization [19–21], these cues are thought to make a negligible contribution to localization in the horizontal plane under normal hearing conditions [11, 35], other than for distinguishing between sources located in front of and behind the listener [13, 36]. There is some evidence, however, that humans with hearing loss in one ear can obtain azimuth information from their intact monaural cues [37–39], while the ability of adult humans [16] and ferrets [40] to adapt with training to a unilateral earplug appears to involve learning to rely more on spectral cues. Thus, cue reweighting may be an important adaptive mechanism irrespective of age.

An involvement of A1 in the flexible processing of spatial cues is consistent with recent work showing that cortical response properties can change depending on the behavioral context in which sounds are presented [41–43]. Our results show that plasticity in the auditory system is also context dependent, with the behavioral reweighting exhibited by juvenile-plugged ferrets closely matched by changes in the spatial sensitivity of cortical neurons. Importantly, however, we observed context-dependent reweighting of auditory spatial cues in cortical neurons even under anesthesia. Unlike previous studies, this makes it extremely difficult to explain our results in terms of cognitive processes like attention and indicates that the dynamic reweighting we observe behaviorally may emerge at relatively low levels of sensory processing. This suggests that context dependence may be a fundamental feature of neural processing, rather than only reflecting the operation of higher-order mechanisms.

Given that spectral cues are thought to play a minimal role in horizontal localization [11, 35], an unexpected finding of our study is that spectral cues can also be recovered, albeit to a far lesser extent, from the behavioral responses of animals with normal hearing. The implication of this, however, is that the auditory system may not need to develop a radically new way of localizing sound following unilateral hearing loss but may instead acquire the ability to leverage mechanisms that are largely latent in normal animals performing the same task. This is consistent with previous evidence for redundancy in the various cues used to localize sound [44]. Because monaural spatial cues are unreliable if the source spectrum is unknown [18], it makes sense to give these cues little weight when binaural cues provide accurate information about source position. But in situations where particular cues provide misleading or degraded information, which can happen either in complex reverberant environments or following impairment of hearing in one ear, it may be better to change the weight afforded to different cues. Auditory localization can therefore be viewed as a process of optimal cue integration, with binaural cues dominating under the acoustical conditions typically experienced by listeners with normal hearing, and increased reliance on spectral cues occurring when comparisons between the ears have been compromised.

In summary, our results show that the auditory system can adapt to an intermittent hearing loss in one ear, and does so by becoming more dependent on the preserved monaural spatial cues provided to the intact ear. Importantly, the context-specific nature of this adaptive plasticity also allows accurate sound localization to be maintained whenever normal acoustical input is available. This is precisely the sort of mechanism that would be required to protect against the long-term deleterious effects associated with otitis media with effusion, a clinical disorder that is extremely common in children and typically produces intermittent periods of hearing loss [9, 10]. More generally, however, our results suggest that it may be possible to reverse the negative effects of abnormal developmental experience by providing intermittent experience of normal sensory input. Consequently, while the context dependence we observe is likely to be critical for maintaining accurate sensory representations in dynamic environments, it may also be possible to leverage these mechanisms for therapeutic benefit.

## Experimental Procedures

Twenty-one ferrets were used in this study, of which ten were raised with an earplug while the remainder were raised normally. The left ear of the juvenile-plugged ferrets was first fitted with an earplug (EAR Classic) between postnatal day 25 (P25) and 29, shortly after the age of hearing onset in this species [45], and was thereafter monitored routinely and replaced as necessary (see Supplemental Experimental Procedures). All procedures were performed under licenses granted by the UK Home Office and met with ethical standards approved by the University of Oxford.

The animals were trained from ∼P150 onward to approach the location of 200 ms broadband noise bursts with either flattened or randomized spectra in order to receive a water reward. Stimuli were presented from one of 12 speakers positioned in the horizontal plane. Reverse correlation maps were constructed from behavioral responses to randomized-spectrum stimuli. A simulated version of this task was also used to assess the performance of monaural and binaural models of sound localization (see Supplemental Experimental Procedures).

Recordings were made under medetomidine/ketamine anesthesia from A1 units in response to VAS stimuli generated from acoustical measurements in each animal [23]. These stimuli recreated the acoustical conditions associated with either normal hearing or an earplug in the left ear and were used to manipulate individual spatial cues independently of one another (see Supplemental Experimental Procedures). We calculated the mutual information (MI) between the spike counts within a window spanning the response of each acoustically driven unit (juvenile-plugged ferrets, n = 260; controls, n = 147) and the virtual azimuth corresponding to each of the available spatial cues. In the case of spectral cues, this was done by collapsing the data across azimuth for the other ear. Estimates of MI were used to calculate a weighting index (WI), which compared the MI values obtained using the spatial cues provided by the nonoccluded right ear (MI_R_) with the sum of the MI values obtained using the other available spatial cues (MI_other_):

$$WI=\frac{MI_{R}-MI_{other}}{MI_{R}+MI_{other}}.$$

## Supplemental Information

Document S1. Figure S1 and Supplemental Experimental Procedures
