# Supplementary material for: Context-Specific Reweighting of Auditory Spatial Cues following Altered Experience during Development
Source: Curr Biol. 2013 Jul 22;23(14):1291–9. doi: 10.1016/j.cub.2013.05.045 (PMC3722484; doi:10.1016/j.cub.2013.05.045)
Supplement: Document S1. Figure S1 and Supplemental Experimental Procedures [file mmc1.pdf]

Current Biology, Volume *23*

Supplemental Information

Context-Specific Reweighting of Auditory Spatial Cues following Altered Experience during Development

Peter Keating, Johannes C. Dahmen, and Andrew J. King

Supplemental Inventory

Figure S1. Location-dependent effects of left-ear occlusion on a sound localization task, related to Figure 1. Illustrates spatial distribution of behavioral responses for each stimulus location tested. Figure 1 summarizes these data as percentages of correct responses.

Supplemental Experimental Procedures. Provides additional details of all experimental procedures.

Supplemental References

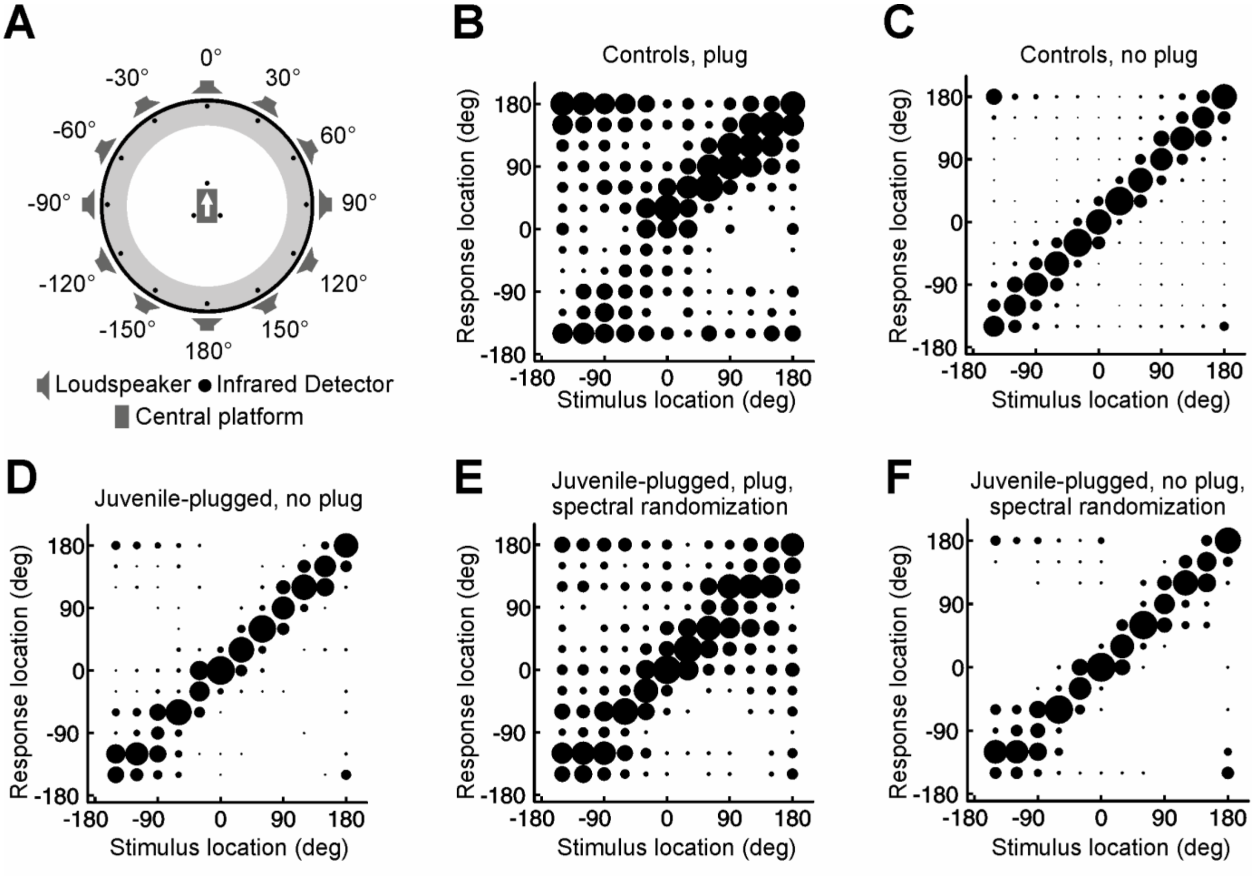

Figure S1. Location-Dependent Effects of Left-Ear Occlusion on Sound Localization in the Horizontal Plane

(A) Schematic of chamber used for behavioral testing. Animals waited at a central platform (■), facing toward 0°, until a sound was presented from one of twelve loudspeakers (◂) situated around the periphery. Responses and readiness for trial initiation were determined using infrared detectors (•).

(B-F) Average joint distributions of stimulus and response location for different groups of animals; the area of the circles represents the proportion of trials for each stimulus-response combination. Azimuth angles as in (A). The stimuli were broadband noise bursts with either a flat (B-D) or randomized (E and F) spectrum. Data are shown for control (B and C) and juvenile-plugged (D-F) animals either in the presence (B and E) or absence (C, D, and F) of a left earplug.

Supplemental Experimental Procedures

General

All procedures were performed under licenses granted by the UK Home Office and met with ethical standards approved by the University of Oxford. General details concerning animal welfare and behavioral testing have been described previously [1]. Briefly, ferrets were housed in small groups within environmentally enriched laboratory cages. Each period of behavioral testing lasted for up to 14 days, during which drinking water was provided through correct performance of the behavioral task and via additional supplements provided at the end of each day. Dry food was made available at all times, and free access to water was given between testing periods.

Earplugs were replaced either with or without sedation (Domitor, 0.1 mg/kg; Pfizer, Walton Oaks, UK). To secure the earplug in place, the concha of the external ear was additionally filled with an ear mold impression compound (Otoform-K2, Dreve Otoplastik, Unna, Germany). The status of both ears was checked routinely using conventional otoscopy and tympanometry, with any accumulation of cerumen removed under sedation. Earplugs were periodically removed so that normal hearing was experienced ~20% of the time, amounting to a total of 3 days in any 15 day period, with periods of non-occlusion evenly spread throughout the diurnal light cycle. This procedure was repeated continuously until the conclusion of the experiments, and enabled a small amount of behavioral testing to be performed in the absence of an earplug. Earplugs were kept in place for at least 4 days prior to terminal electrophysiological experiments, and were only removed immediately prior to surgery. In general, juvenile-plugged ferrets were tested behaviorally without an earplug on < 3 separate occasions, each of which consisted of multiple test sessions spread over 1-3 days. In all cases, no obvious differences were observed between data obtained on different days following earplug removal.

Behavioral Testing

Behavioral testing was performed in a sound-proof chamber, within which was situated a custom-built circular mesh enclosure with a diameter of 1.25 m. Animals initiated each trial by standing on a platform located in the middle of the testing chamber and licking a spout positioned at the front of this platform for a variable delay, following which a stimulus was presented from one of 12 loudspeakers (FRS 8; Visaton, Crewe, UK) equally spaced around the periphery of the enclosure (Fig. S1A). Behavioral responses and trial initiation were monitored using infrared detectors, with experimental contingencies controlled using Matlab (The MathWorks, Natick, MA). Correct responses were rewarded with water delivered via spouts situated beneath each loudspeaker. Incorrect responses were followed by correction trials on which the same stimulus was presented. Persistent failure to respond correctly was followed by ‘easy trials’ on which the stimulus was repeated continuously until the animal made a response. Data from correction and easy trials were excluded from all analyses.

Behavioral test stimuli consisted of 200-ms bursts of randomly-generated broadband noise that were generated with a sampling rate of 97.6 kHz, band-pass filtered (0.5-30 kHz), and rendered spectrally flat by compensating for the transfer function of each speaker. Although the hearing range in the ferret extends from ~36 Hz to 44 kHz at moderately high sound levels [2], thresholds become substantially elevated at frequencies > 30 kHz, making this species less sensitive to any spectral cues that might be available at such high frequencies. In addition, it can be very difficult to adequately control loudspeaker output at frequencies that lie outside the range

0.5-30 kHz. Consequently, previous studies in the ferret have tended to use stimuli with a high-frequency cutoff of 30 kHz [3,4]. To facilitate comparison with previous work, we therefore employed very similar stimuli.

Cosine ramps (10 ms duration) were applied to the onset and offset of each stimulus, and the level was varied randomly between 56 and 84 dB SPL in increments of 7 dB. Spectral randomization was achieved by adding a vector to the logarithmic representation of the source spectrum, with the type of randomization defined by the nature of this vector.

In some experiments, this randomization vector was created from Gaussian noise that was low-pass filtered to remove all energy at frequencies >3 cycles/octave, thereby ensuring that the randomization vector changed relatively smoothly as a function of frequency. The resulting vector was normalized so that it had a mean of zero and an RMS equal to σ. On each trial, σ was set to either 1.25 or 10 dB in a randomly interleaved fashion. When σ equaled 1.25 dB, the stimulus spectrum was relatively stable across trials. We therefore refer to these as flat-spectrum stimuli to distinguish them from stimuli with σ equal to 10 dB, which exhibited a much greater degree of spectral variability across trials. Only incorrect responses to flat-spectrum stimuli were followed by correction and easy trials.

In other experiments, the randomization vector was first separated into bands $1/5^{th}$ of an octave wide, with the level of each band set independently to values drawn randomly from a normal distribution with a mean of zero and a standard deviation σ. In each session, the same σ was used on all trials, with values of 0, 10 and 15 dB used in different sessions. Incorrect responses were always followed by correction and easy trials, irrespective of stimulus type.

Behavioral Analyses

Confidence intervals at the 95% level were estimated empirically for different measures using 10,000 bootstrapped samples, each of which was obtained by re-sampling with replacement from the original data. These samples were then used to construct bootstrapped distributions of the desired measure, from which confidence intervals were derived [5].

A bootstrap procedure was also used to assess the significance of group differences. First, the difference between two groups was measured using an appropriate statistic (e.g. difference in means). The data from different groups were then pooled and re-sampled with replacement to produce two new samples, and the difference between these samples was measured using the same statistic as before. This procedure was subsequently repeated 10,000 times, which provided an empirical estimate of the distribution that would be expected for the statistic of interest under the null hypothesis. This bootstrapped distribution was then used to derive a P value for the difference observed in the original sample. In some cases (response time analyses and Fig. 4E), data were pooled across animals before applying the re-sampling procedure described above to the data obtained on individual trials. In all cases, conventional parametric and non-parametric statistical tests were also performed and produced very similar results to those obtained by bootstrapping.

To determine significance levels for the effects of spectral randomization, we used mixed-effects logistic regression with degree of randomization and testing period (plug, no plug, replug) as fixed effects, and subject as a random effect. Appropriate post-hoc tests were then conducted for comparisons of interest. This approach took advantage of the fact that, whilst relatively few subjects were tested, many observations were made for each subject.

Reverse correlation maps (RCMs) were constructed from behavioral responses made to randomized-spectrum stimuli (~3,000 trials for each animal) by computing the mean stimulus

spectrum for all trials on which an animal responded to a specific location, with the resulting spectrum subsequently band-pass filtered so that it contained energy from 0.5-3.0 cycles/octave. This was done because sound localization is relatively insensitive to very rapidly- or slowly-changing spectral features [6,7]. This analysis was repeated for each response location and the spatial mean was subtracted from each of the identified spectra.

To estimate the magnitude of spectral features present in each RCM, a threshold was applied, with values < 1.5 SD away from the overall mean set to 0. The feature strength (Fig. 3C) was then defined as the unsigned spatial mean of the resulting RCM. The feature strength was separated into frequency bands 0.5 octaves wide and averaged within each band. Because feature strength is calculated using the unsigned spatial mean, it will necessarily produce positive values, even in situations where there is no relationship between the stimulus spectrum and the behavioral response. To remove this bias toward positive values, we therefore shuffled the data and estimated the feature strength values for the shuffled data. This provided a measure of bias, which we then subtracted from the original (and unshuffled) estimate of feature strength. This procedure ensured that the feature strength was very close to zero in situations where the stimulus spectrum had no influence on the behavioral response. This problem of bias, as well as the solution adopted here, is directly analogous to that experienced with information theoretic measures [8].

To estimate the spectral cues that are typically available to the ferret, we used a set of directional transfer function (DTF) measurements previously obtained in a separate study from 13 ferrets, none of which were used in the present study [9]. To facilitate comparison between these DTFs and the behavioral RCMs, we selected DTF filters corresponding to the stimulus locations used for behavioral testing, separated each spectrum into frequency bands $1/20^{th}$ of an octave wide, and averaged the energy in each band. The resulting spectra were then band-pass filtered so that they contained energy only from 0.5-3.0 cycles/octave and averaged across animals. These averaged spectral cues were then compared with the averaged RCMs obtained from both juvenile-plugged animals and controls.

Modeling

To assess the performance of monaural and binaural models of sound localization, we simulated a behavioral task using the same stimuli and task design as described above. Following stimulus generation, the stimulus was positioned in virtual acoustic space (VAS) by convolving it with directional filters appropriate to a particular location, thereby providing virtual acoustical inputs for each ear of the model. The directional filters used were taken directly from the DTF of an individual ferret [9].

On each trial i, the monaural model was only provided with the spectral input available to the right ear ($\omega_{R,i}$). The model then computed the correlation between $\omega_{R,i}$ and the spectra of the directional filters associated with the right ear at each location j ($Z_{R,j}$), and selected a response (r_i) by determining the location at which this correlation function achieved a maximum:

$$r_i = \underset{j}{\operatorname{argmax}}\, \rho(\omega_{R,i}, Z_{R,j})$$

The binaural model made additional use of the spectral input provided to the left ear ($\omega_{L,i}$), thereby allowing responses to be based on frequency-dependent ILDs:

$$r_i = \underset{j}{\operatorname{argmax}}\, \rho\left(\log\frac{\omega_{R,i}}{\omega_{L,i}}, \log\frac{Z_{R,j}}{Z_{L,j}}\right)$$

Neurophysiological Recordings

Recordings were carried out under anesthesia in order to provide the stability needed for presenting large stimulus sets via earphones a sufficient number of times to obtain estimates of mutual information (MI) between the spiking responses of the neurons and each localization cue. This also meant that we could eliminate changes in the arousal level or attentional modulation as factors contributing to any differences observed between the control and juvenile-plugged ferrets or between the normal hearing and virtual earplug conditions.

Briefly, animals were anesthetized with Domitor and ketamine hydrochloride (Ketaset; Fort Dodge Animal Health, Southampton, UK), and given a subcutaneous dose of atropine sulphate (0.01 mg/kg; C-Vet Veterinary Products, Leyland, UK). Anesthesia was maintained using a continuous intravenous infusion of Domitor (22 µg/kg/hr) and ketamine (5 mg/kg/hr) in 0.9% saline supplemented with 5% glucose and dexamethasone (0.04 mg/kg/hr Dexadreson; Intervet UK, Milton Keynes, UK). Animals were intubated and artificially ventilated with oxygen. End-tidal CO_2 and heart rate were monitored, and body temperature was maintained at ~38.5°C.

The animal was placed in a stereotaxic frame fitted with blunt ear bars, and the skull was exposed. A stainless steel bar was then attached to the caudal midline of the skull and secured in place using a combination of bone screws and dental cement. The displaced tissue was then reattached to the skull using veterinary adhesive (Vetbond, SM Animal Care Products, 3M Animal Care Products, St. Paul, MN), allowing the ears to be returned to their natural position. The stereotaxic frame was subsequently removed and the animal was moved to an anechoic chamber.

Damped polythene probe tubes were passed through the caudal walls of the ear canals, secured in place, and connected to condenser microphones (MKE 2PC, Sennheiser UK Ltd, High Wycombe, UK), the output of which was amplified (DMP3, M-Audio, Bucks, UK) and digitized at 97.6 kHz (RP2; Tucker-Davis Technologies (TDT), Alachua, FL). Acoustical signals were recorded for 24 different azimuths, each of which was separated by 15°, using 512-point Golay codes [10] generated in Matlab, converted to analog signals (RP2), amplified (RA150; Alesis, Cumberland, RI), and presented using a loudspeaker (KEF T27) mounted on a vertical motorized hoop with a radius of 65 cm.

Once the acoustical recordings were complete, the temporal muscles were removed and bilateral craniotomies were made over the auditory cortex. The overlying dura was removed, and silicone oil was applied to the cortical surface. In each hemisphere, a single-shank silicon probe (Neuronexus Technologies, Ann Arbor, MI) with 16 recording sites spread over a length of 1.5 mm was lowered into A1. The position of the probe was confirmed by measuring frequency response areas, which were used to derive the characteristic tonotopic gradients that delineate cortical fields in the ferret [11].

Neural signals were band-pass filtered (500 Hz – 3 kHz), amplified, and digitized (25 kHz) using TDT System 3 processors. Stimuli were generated in Matlab, amplified (TDT headphone amplifier) and presented via earphones (Panasonic, RP-HV298, Bracknell, UK) that were situated at the entrance to each ear canal. Action potentials were extracted in Brainware from a mixture of single units and small multi-unit clusters, which were pooled for subsequent analyses to increase statistical power. All further data analyses were carried out using Matlab.

VAS stimuli were produced by replicating the spectra associated with free-field stimuli using appropriately filtered closed-field stimuli. The filters were obtained by dividing the Fourier transform of the microphone response to free-field stimulation by that to closed-field stimulation

and generating a minimum-phase filter with the appropriate transfer function. The resulting filters were used to recreate VAS stimuli for normal hearing conditions. In addition to the filters used to replicate free-field stimuli under normal hearing conditions, we simulated a unilateral hearing loss using a second set of filters in which the filters for the left ear incorporated an additional low-pass filter (Fig. 5B) and a delay of 110 μs. The precise nature of this filter was based on previous ear-plugging studies in ferrets [12], and was qualitatively very similar to more recent measurements in chinchillas [13] and humans [14].

For each type of VAS, we generated a set of stimuli in which the spectral cues were manipulated independently and concurrently for each ear. These cues corresponded to a set of locations in azimuth that included [-45°, 0°, 45°, 90°, 135°], where positive values indicate positions in the right hemifield and 0° denotes the position immediately to the front. All stimuli were repeated 30 times and presented at 84 dB SPL. ILD sensitivity was measured separately by varying the ILD between ±14 dB in increments of 7 dB (prior to any effects of a virtual earplug). For the purposes of assessing ILD sensitivity, spectra in each ear were set to values corresponding to 0° azimuth.

Neurophysiological Analyses

The mutual information (MI) between two discrete variables X and Y is defined as:

$$MI(X;Y) = \sum_{y \in Y} \sum_{x \in X} p(x,y) log\left(\frac{p(x,y)}{p(x)p(y)}\right)$$

where p(x,y) denotes the joint probability distribution of X and Y and p(x) and p(y) are respectively the marginal probability distributions of X and Y. Estimates of MI were debiased using a bootstrap procedure, which computes the MI between 500 random pairings of X and Y and subtracts the median of this distribution from the original estimate of MI [15]. Observed distributions of both the MI between spike count and virtual azimuth corresponding to each spatial cue and the weighting index (WI) values violated the assumptions of standard parametrical tests for statistical significance. The significance of factor effects and their interactions were therefore assessed using permutation tests [16]. This involved randomly permuting observations across different factors and calculating an F statistic for each factor and interaction (i.e. the proportion of variance explained relative to the proportion of unexplained variance). This procedure was repeated many times in order to assess the percentage of repetitions that produce F values greater than those obtained for the non-permuted data. This percentage then provided an estimate of the P values associated with each effect under the null hypothesis. Precise details of the permutation procedure used have been described elsewhere [16]. Comparisons between conditions were also made using Kruskal-Wallis tests and appropriate post-hoc testing.

Supplemental References

1. Nodal, F.R., Bajo, V.M., Parsons, C.H., Schnupp, J.W., and King, A.J. (2008). Sound localization behavior in ferrets: comparison of acoustic orientation and approach-to-target responses. Neuroscience *154*, 397-408.
2. Kelly, J.B., Kavanagh, G.L., and Dalton, J.C. (1986). Hearing in the ferret (Mustela putorius): thresholds for pure tone detection. Hear Res *24*, 269-275.
3. Carlile, S., and King, A.J. (1994). Monaural and binaural spectrum level cues in the ferret: acoustics and the neural representation of auditory space. J Neurophysiol *71*, 785-801.
4. Nodal, F.R., Kacelnik, O., Bajo, V.M., Bizley, J.K., Moore, D.R., and King, A.J. (2010). Lesions of the auditory cortex impair azimuthal sound localization and its recalibration in ferrets. J Neurophysiol *103*, 1209-1225.
5. Davison, A.C., and Hinkley, D.V. (1997). Bootstrap Methods and Their Application (Cambridge: Cambridge University Press).
6. Kulkarni, A., and Colburn, H.S. (1998). Role of spectral detail in sound-source localization. Nature *396*, 747-749.
7. Hofman, P.M., and Van Opstal, A.J. (2002). Bayesian reconstruction of sound localization cues from responses to random spectra. Biol Cybern *86*, 305-316.
8. Montgomery, N., and Wehr, M. (2010). Auditory cortical neurons convey maximal stimulus-specific information at their best frequency. J Neurosci *30*, 13362-13366.
9. Schnupp, J.W., Booth, J., and King, A.J. (2003). Modeling individual differences in ferret external ear transfer functions. J Acoust Soc Am *113*, 2021-2030.
10. Schnupp, J.W., Mrsic-Flogel, T.D., and King, A.J. (2001). Linear processing of spatial cues in primary auditory cortex. Nature *414*, 200-204.
11. Bizley, J.K., Nodal, F.R., Nelken, I., and King, A.J. (2005). Functional organization of ferret auditory cortex. Cereb Cortex *15*, 1637-1653.
12. Moore, D.R., Hutchings, M.E., King, A.J., and Kowalchuk, N.E. (1989). Auditory brain stem of the ferret: some effects of rearing with a unilateral ear plug on the cochlea, cochlear nucleus, and projections to the inferior colliculus. J Neurosci *9*, 1213-1222.
13. Lupo, J.E., Koka, K., Thornton, J.L., and Tollin, D.J. (2010). The effects of experimentally induced conductive hearing loss on spectral and temporal aspects of sound transmission through the ear. Hear Res *272*, 30-41.
14. Kumpik, D.P., Kacelnik, O., and King, A.J. (2010). Adaptive reweighting of auditory localization cues in response to chronic unilateral earplugging in humans. J Neurosci *30*, 4883-4894.
15. Panzeri, S., Senatore, R., Montemurro, M.A., and Petersen, R.S. (2007). Correcting for the sampling bias problem in spike train information measures. J Neurophysiol *98*, 1064-1072.
16. Manly, B.F.J. (2007). Randomization, Bootstrap, and Monte Carlo Methods in Biology, Third Edition (London: Chapman & Hall).
